# Delivering genome sequencing for rapid genetic diagnosis in critically ill children: parent and professional views, experiences and challenges

**DOI:** 10.1038/s41431-020-0667-z

**Published:** 2020-06-19

**Authors:** Melissa Hill, Jennifer Hammond, Celine Lewis, Rhiannon Mellis, Emma Clement, Lyn S. Chitty

**Affiliations:** 1 North Thames Genomic Laboratory Hub, Great Ormond Street Hospital for Children NHS Foundation Trust, London, UK; 2 Genetics and Genomic Medicine, UCL Great Ormond Street Institute of Child Health, London, UK; 3 Clinical Genetics and Genomic Medicine, Great Ormond Street Hospital for Children NHS Foundation Trust, London, UK

**Keywords:** Health services, Social sciences

## Abstract

Rapid genomic sequencing (RGS) is increasingly being used in the care of critically ill children. Here we describe a qualitative study exploring parent and professional perspectives around the usefulness of this test, the potential for unintended harms and the challenges for delivering a wider clinical service. The Rapid Paediatric Sequencing (RaPS) study offered trio RGS for diagnosis of critically ill children with a likely monogenic disorder. Main and actionable secondary findings were reported. Semi-structured interviews were conducted with parents of children offered RGS (*n* = 11) and professionals (genetic clinicians, non-genetic clinicians, scientists and consenters) (*n* = 19) by telephone (parents *n* = 10/professionals *n* = 1) or face-to-face (parents *n* = 1/professionals *n* = 18). We found that participants held largely positive views about RGS, describing clinical and emotional benefits from the opportunity to obtain a rapid diagnosis. Parental stress surrounding their child’s illness complicates decision making. Parental concerns are heightened when offered RGS and while waiting for results. The importance of multidisciplinary team working to enable efficient delivery of a rapid service was emphasised. Our findings give insight into the perceived value of RGS for critically ill children. Careful pre-test counselling is needed to support informed parental decision making. Many parents would benefit from additional support while waiting for results. Education of mainstream clinicians is required to facilitate clinical implementation.

## Introduction

Genetic disorders are a leading cause of morbidity and mortality in both neonatal and paediatric intensive care units (NICUs/PICUs) [1–3]. Rapid genomic sequencing (RGS) can be invaluable for directing patient management, including medical or surgical treatment options or, in some cases, decision making around palliative care [4–13]. RGS can also prevent the need for diagnostic interventions that could be invasive or painful [9] and there is evidence that it may help in avoiding costs associated with additional interventions and in reducing length of stay [10].

While there is great potential for genomic sequencing to have a beneficial impact on how genetic conditions are diagnosed and treated, integrating  these tests into clinical care involves multiple considerations around how the tests are offered, what results should be returned, implications for other family members, data protection, and ensuring informed consent [14]. For RGS in paediatric critical care additional challenges include supporting parents needing to process complex information and make informed decisions during a distressing and time-pressured period [12]. There are also concerns around costs, changing expectations for professional roles and potential burdens on laboratories [10].

Whilst there is a growing body of research looking at stakeholder views and experiences of genomic sequencing for the diagnosis of rare diseases in children, very few studies focus on offering RGS to critically ill children. Studies in the US [15] and Canada [16] have examined parental experiences of RGS in the NICU only. Professional attitudes have been explored in the US [17–19] and Australia [20] and consider neonatal and paediatric settings. Here we describe qualitative research conducted with UK professionals and parents of critically ill children, across neonatal and paediatric age groups, offered RGS in a UK research study conducted within an NHS clinical practice setting [9]. We have used qualitative research to gather and integrate the views and experiences of parents and professionals to gain an in-depth understanding of offering RGS to children in critical care settings from multiple perspectives. Our study aimed to consider decision making, psychosocial impacts, potential harms and challenges for service delivery with a view to informing policy and practice for national clinical implementation of RGS in in the new NHS Genomic Medicine Service in England and other settings.

## Materials and methods

### Setting

The Rapid Paediatric Sequencing (RaPS) study was initiated at Great Ormond Street Hospital for Children NHS Foundation Trust (GOSH), a tertiary children’s hospital specialising in rare diseases, in August 2015. The RaPS study offered RGS to critically ill children seen in NICU, PICU or cardiac intensive care units who were thought likely to have a monogenic disorder and genetic diagnosis could inform patient management [9]. RGS was performed in trios (patient and parents) and variants assessed as “pathogenic” (known to affect function) or “likely pathogenic” (likely to affect function) and contributing to the individual’s condition were reported. Parents were given the choice of opting in or out of the return of medically actionable secondary findings, guided by the American College of Medical Genetics and Genomics recommendations [21]. Mestek-Boukhibar et al. [9] describes the development of the RGS workflow and the testing of the first 24 critically ill children who took part in the RaPS study. At the time of RGS, the children in the RaPS study had a mean age of 15.86 months (range 7 days to 13 years and 2 months) with a median age of 2.5 months. RGS led to a molecular diagnosis in ten (42%) cases and in three of these ten (30%) the diagnosis had an immediate impact on the child’s clinical management [9].

### Recruitment

Two participant groups were recruited: (1) Parents of children offered RGS as part of the RaPS study and (2) professionals (genetic clinicians, non-genetic clinicians, genetic scientists and research consenters) involved in offering and delivering RaPS. Parents participating in the RaPS study between one and  four years ago, aged over 18 years and able to communicate in English were identified by genetic clinicians at GOSH. Following discussion with relevant clinicians to ensure it was appropriate to contact parents, they were mailed the participant information. If there was no response after 2 weeks a clinician (RM) telephoned potential participants to discuss the study. If interested in taking part, parents were contacted by the qualitative researchers (JH, CL or MH) to organise an interview time. Parents were offered a £10 gift voucher in appreciation of their time. Professionals actively involved in offering RGS or delivering RGS through the RaPS study were identified by the research team and invited to participate by MH or CL via email. All participants were given the option of face-to-face or telephone interviews.

### Interviews

Semi-structured interviews were conducted face-to-face or via telephone by researchers experienced in conducting interviews on sensitive topics (MH, CL and JH). Topic guides were initially drafted by MH and CL, then revised following feedback from EC, LSC and MB-G; genetics clinicians with experiential knowledge of RGS. Topic guides explored; 1. Experiences of the RaPS study. 2. Recollections of the information given about RGS and the consent process. 3. The findings of RGS and the impact (including benefits and harms). 4. General thoughts on offering RGS in critical care settings. 5. Challenges for service delivery (professionals only). Standard demographic questions were included for both parents and professionals (Table 1).

**Table 1:** Participant characteristics.

Characteristic	Parents (*N* = 11)	Professionals (*N* = 19)
Gender		
Female	3	8
Male	8	11
Age group (years)		
21–30	5	1
31–40	5	4
41–50	1	9
51–60	0	3
>60	0	2
Ethnicity		
Asian British	7	Not applicable
White British	3	
Other	1	
Highest qualification		
No qualification	1	Not applicable
High school	5	
Degree or equivalent	6	
Age of child at time of RGS	*N* = 10	
≤1 month	4	Not applicable
1–9 months	3	
1–2 years	1	
10–15 years	2	
RGS findings for child	*N* = 10	
Diagnosis	5	Not applicable
Partial diagnosis	2	
Variant of uncertain significance	1	
No findings	2	
Current role	Not applicable	
Scientist		3
Genetic consultant		5
Medical doctor trainee (consenter for the RaPS study)		1
Non-genetic paediatric consultant		10
* Cardiology*		1
* Endocrinology*		1
* Immunology*		1
* Intensive care*		1
* Metabolics*		2
* Nephrology*		1
* Neurology*		2
* Neuromuscular*		1
Years in current role	Not applicable	
2–10		8
11–20		8
21–30		2
>30		1

### Data analysis

Interviews were digitally recorded, transcribed verbatim and anonymised. Data were analysed using the principles of thematic analysis [22] and combined inductive and deductive approaches [23] as themes were drawn from the topic guides, which reflected the existing literature, clinical experience and our study aims, and emerged from the empirical data. NVivo version 12 (QSR International, Pty Ltd, Australia) was used to facilitate coding and data analysis. Data collection and analysis were performed concurrently. Draft codebooks were developed by MH for the professional and parent interviews based on the topic guides, MH and JH then independently coded the same transcript and added further codes. The two researchers then independently coded two further transcripts. Discrepancies were discussed and revised codebooks were developed to act as a guide for the coding of the remaining transcripts. The overarching themes did not change greatly between the draft and final codebooks, but many additional codes, that were later refined into sub-themes, were included, particularly for the parent codebook as this reflected individual experiences. Emergent themes and sub-themes were reviewed and revised by MH, JH and CL. Parent and professional interviews were initially treated as two separate data sets that were analysed independently. When compared, the themes from the two groups were found to overlap and have been combined into a single narrative. Any topics or themes emerging from only one participant group are noted in the results.

## Results

Of the 40 families enrolled in the RaPS study who were eligible for our interview study, ten were not contacted as they were not fluent in English, were not living in the UK or clinicians felt contact could potentially cause further distress. Parents of the remaining 30 children were invited to participate. Two declined, three could not be contacted by telephone and 15 did not respond. A total of 11 parents (ten families) took part in an interview (response rate 33%). Of 27 professionals invited by email to participate, six did not respond, two declined and 19 participated (response rate: 70%). Professional interviews were conducted between September 2018 and May 2019 (18 in person, one by phone) and lasted between 15 and 56 min (median = 38 min). Parent interviews were conducted between December 2018 and November 2019 (one face-to-face, ten by phone) and lasted between 20 and 62 min (median = 38 min). Participant characteristics are described in Table 1. At the time of RGS, the majority of children of the parents we interviewed were aged under 9 months (7/10), the other three children were; one, 12 and 13 years old. Five children obtained a diagnosis through RGS; Sotos syndrome (MIM 606681), vascular Ehlers–Danlos syndrome (MIM 120180), Holt–Oram syndrome (MIM 142900), Joubert syndrome (MIM 612013) and multiple congenital anomalies-hypotonia-seizures syndrome (MIM 615398). In two of these cases, diagnoses of vascular Ehlers–Danlos syndrome and Sotos syndrome, there was an immediate impact on clinical management. Two children received a partial diagnosis through RGS; blepharophimosis ptosis and epicanthus inversus syndrome (MIM 110100) and STING-associated vasculopathy with onset in infancy (MIM 615934). A variant of uncertain significance was reported for one child and two children had no reported findings.

### Positive perceptions of offering RGS to critically ill children

Parents and professionals held very positive views about RGS being available in paediatric critical care settings which was expressed through words like “lucky”, “pleased” and “privileged”. Parents’ primary motivation for consenting to RGS was to obtain information about the cause of their child’s illness, prognosis and treatment options. As one parent explained, “you do whatever you need to do, in order to get a picture, a conclusion of what’s gone on” (Parent-6, child diagnosed with Sotos syndrome). Parents also described the seriousness of the illness and a need for certainty as reasons, with other motivations including wanting information for future family planning and altruism; “anything you can do to help your child at that point in time and other children, it’s got to be worthwhile” (Parent-2, child partially diagnosed (blepharophimosis, ptosis, and epicanthus inversus syndrome).

#### Clinical benefits

Parents and professionals valued the clinical benefits of RGS for critically ill children such as rapid testing of multiple genes to obtain a diagnosis or rule out conditions (Table 2: Q1). RGS allows greater certainty and clarifies possible comorbidities and prognosis (Table 2: Q2) and the broad scope of RGS was seen as particularly useful for complex patients with non-specific symptoms (Table 2: Q3 and Q4). Other clinical benefits discussed by parents and professionals were linked to having a diagnosis, such as gaining information for future pregnancies and the wider family (Table 2: Q5). Benefits for research and learning about rare conditions that will bring clinical benefits in the future were also noted (Table 2: Q6). Professionals discussed cases where RGS findings made a major difference to patient management (Table 2: Q7) or informed decisions to initiate palliative care (Table 2: Q8). The importance of ruling out conditions was notably valued by immunology teams wanting to know if the child had any variants that preclude transplant (Table 2: Q9). The potential to reduce the number of potentially painful or invasive investigations if a diagnosis is rapidly identified was highlighted (Table 2: Q10). It was also noted that when a diagnosis is obtained “immediately you release a lot of resources” which may reduce costs.

**Table 2:** Perceived clinical and psychosocial benefits of RGS in critical care settings.

Topic	Illustrative quotes
Clinical benefits	
Rapidly check multiple genes with a single test	Q1: “I think the main benefit is that the differential diagnosis is very wide and broad…you can test for many things with one test, you can cast your net wide, you don’t have to be as specific and the timeframe in which you can get the results back is quite impressive.” Professional-7, medical doctor trainee, consenter for RaPS
Clarify prognosis	Q2: “And it was important for us to know firstly what she has and what that means for her, in terms of her prognosis and the care she might need long term” Parent-1, child diagnosed with Joubert syndrome
Useful test for complex conditions with non-specific symptoms	Q3: “So the genetics was really important because they realised that the breathing problems was not a syndrome…Yeah, you’re finally see the whole thing, you could like be sure what’s going on, so yes looking to this way the genetics make a huge difference on his treatment and how the doctors decide to do anything” Parent-7, child diagnosed with Holt–Oram syndrome Q4: “I think the kids, it’s just the ones that are the sickest…the clinical team are lost, what are we going to do next, well let’s see if we can find the gene and if we can find the gene that might give us a clue on how we can go next.” Professional-9, scientist
Information for future pregnancies and for the wider family	Q5: “And I guess we also had another eye for, you know we were talking about genetics, you know, thinking about you know, future children. If there was anything genetic it would be good to know for the next time.” Parent-3, child diagnosed with Joubert syndrome
Benefits for research	Q6: “To diagnose children, to find other children with the same thing, maybe in a different country, I don’t know, you know, so you can work together and get a cure or find out what they’re doing to manage it or what other people are doing to manage it.” Parent-4, child not diagnosed
Guides treatment and management	Q7: “So once you know that’s the diagnosis, you can look at the list of ways that condition manifests itself and the things that those children are at risk of and so it gives you a much better idea of what kind of monitoring they might need in the future, what teams would need to be involved, what outpatient care they might need and that kind of thing” Professional-7, medical doctor trainee, consenter for RaPS
Informs decisions around palliative care	Q8: “I mean, this child doesn’t have a treatment, we have to think about going down the palliation pathway and making that as nice as possible… and I think palliation works best if you can get in early so [RGS is] actually as helpful for conditions where there’s no treatment as it is for conditions where there is a treatment.” Professional-2, non-genetic clinician
Ruling out conditions	Q9: “It’s been more useful knowing that patients haven’t got a defined subset of genes, because then we could give a bone marrow transplant and kill them instantly. I think there was true benefit to not getting a diagnosis, in that we progressed to transplant.” Professional-5, non-genetic clinician
Reduces the number of investigations	Q10: “If this child did not have a diagnosis up to now, we would have put her under further MRIs and maybe further blood tests or urine tests but at least we got an answer from the rapid sequence. That kind of stopped the clock of the investigations…The second thing of course, which goes again with a quick diagnosis is that immediately you release a lot of resources that otherwise would have gone for this child or for repeat examinations.” Professional-12, non-genetic clinician
Psychosocial benefits	
Opportunity to gain certainty in a very uncertain setting	Q11: “So when they got his results, it was like ‘OK we know what we’re doing and now we know where and when and what we need to do’, so it was the end of a lot of questions.” Parent-7, child diagnosed with Holt–Oram syndrome Q12: “These children are very ill…and we’re getting involved often because things aren’t improving. And so I think the parents have got the idea of a lot of uncertainty and you can deal with really difficult situations when there’s more certainty than you can when there’s uncertainty.” Professional-17, genetic clinician
Relief	Q13: “It was a mixture of relief, and sadness as well. [Long pause] Relief to know what it was, sadness to know that he’s got a condition. Also, you kind of take the positive out of it, he's got a condition that hopefully he should fully recuperate from it.” Parent-6, child diagnosed with Sotos syndrome Q14: “It was quite a relief, quite emotional [to have a condition ruled out], because it was quite difficult at the time to accept that and it was a big worry for us… Because it would have affected her life, no doubt, quite badly.” Parent-2, child partially diagnosed with Blepharophimosis, ptosis, and epicanthus inversus syndrome Q15: “When you kind of have like a final stamp on the diagnosis and you can confirm that with the family… then of course you can have devastation but many of the times also have relief actually.” The parents saying to me actually at least it is a closure, it is a final closure for them so it is a relief.” Professional-12, non-genetic clinician
Reassurance	Q16: “I think it’s also we’re dealing with very fraught situations and I think it gives some comfort to the families that you’re doing all that you can in such a difficult situation and, in some ways, that’s more important than the diagnosis.” Professional-17, genetic clinician Q17: “But it was helpful for the parents to know that what the baby had was lethal, that nothing more could have been done to have kept the baby alive.” Professional-1, genetic clinician
Cuts short the diagnostic odyssey	Q18: “I’ve met several families since doing the testing and finding out results who haven’t found out for many years after and they’ve spent many years not knowing. It just makes me realise in hindsight how good that was for us to have had that so early.” Parent-1, child diagnosed with Joubert syndrome
Helps emotionally to get a diagnosis	Q19: “And I think for parents who really don’t know what’s going on, it really does help with just emotionally being able to understand what’s going on and seeking out the right emotional support to help you, enable you in dealing with all of that as well. Just knowing what your child has enables you to talk to other parents who have similar children suffering with similar conditions and just knowing you’re not on your own, especially when you’re looking at rare genetic conditions, where you only have a handful of people.” Parent-1, child diagnosed with Joubert syndrome
Helps emotionally to rule out conditions	Q20: “So we know now genetically, that there is no obvious medical conditions within her make up. So I think that side of things, because there’s stuff you can rule out, which does help you move forward a little bit, it’s something you can just put to the side, and almost forget about really. So yeah, regardless of whether they find something or not, I think it’s nice just knowing, yes.” Parent-11, child not diagnosed

#### Psychosocial benefits

A key psychosocial benefit recognised by parents and professionals was the opportunity for parents to gain certainty in a very uncertain setting (Table 2: Q11 and Q12). Parents felt relief when there was a diagnosis, and when life-limiting conditions had been raised and then ruled out (Table 2: Q13 and Q14). Professionals noted parents can feel relief even when it is a devastating diagnosis as there is closure (Table 2: Q15). Reassurance was reported by professionals as a potential psychosocial benefit for parents, regardless of RGS findings, as “everything has been done to understand what’s wrong” and their child “had the right and the best testing” (Table 2: Q16). One professional also noted that parents maybe reassured when a lethal condition is diagnosed, as it becomes clear there was nothing more they could have done (Table 2: Q17). Other psychosocial benefits of having a diagnosis described by parents and professionals included cutting short the diagnostic odyssey (Table 2: Q18), finding it helpful to understand what is going on and being able to seek appropriate support (Table 2: Q19).

For parents who received uncertain, partial or no findings for their child, emotional benefits of ruling out conditions were also seen. One parent noted that although a diagnosis was not found, being able to rule some conditions out allowed her to “move forward a little bit” (Table 2: Q20).

### Potential negative impacts for parents

#### Projecting the future

When describing the time of being offered RGS, parents discussed being very anxious about their child’s health and some spoke about it being “scary” to be offered RGS, as this added weight to their existing concerns (Table 3: Q1). Parents were often told multiple potential causes for their child’s illness and described being worried about the possible findings and what it would mean for the future (Table 3: Q2). One parent said that her only concern about RGS was “that it might come back that he’s terminally ill” and had mixed feelings about obtaining a diagnosis (Table 3: Q3). In addition, one professional raised the concern that waiting for RGS findings could be an additional worry in the background for parents dealing day-to-day with a critically ill child (Table 3: Q4).

**Table 3:** Potential negative impacts for parents arising from offering RGS in critical care settings.

Topic	Illustrative quotes
Projecting the future	
It can be confronting to be offered RGS	Q1: “So for me, it was a very scary thing to think oh my God, why are they trying to find something wrong with him? Yeah, because—I just kept thinking oh my God, do they think it’s going to be a neurological issue? Are they going to find something to do with his communication issues? Are they going to say ah he’s got a physical element that you know, is related to a symptom?” Parent-8, child partially diagnosed with STING-associated vasculopathy with onset in infancy
Parents face the possibility of a devastating diagnosis	Q2: “It needed to be done to rule things out but we did feel that perhaps she didn’t have this condition, and that there were explanations for her appearance at that point in time. She was quite unwell. But that was tough for us but then it needed to, the testing needed to happen…That was one of the difficult times that sort of stands out a bit…We needed to say yes to it, which was difficult…It did affect [my wife], worrying about this [condition], and then when it was found later on that she didn’t have the disease she was quite emotional at that time. Yeah that did upset her quite a bit.” Parent-2, child partially diagnosed with Blepharophimosis, ptosis, and epicanthus inversus syndrome
Mixed feelings about obtaining a diagnosis	Q3: “It’s hard because half of you wants to find something but then the other half you don’t want to, you know, I just don’t know how to feel about it. Are you better off not knowing if it’s going to be bad news? Or…I don’t know…I don’t know! Obviously I want to know if it’s treatable and if it can be treated then that’s great, but then if I find out that it’s something that’s terminal…then it’s not so good is it.” Parent-4, child not diagnosed
RGS another worry in the background	Q4: “Some people don’t worry about that at all and they forget almost. In fact some people genuinely forget that they’ve had the test because there’s so much going on in their lives, lots of the other things are far more pressing. But some people just having that in the background, it’s another thing that they worry about. And it’s very, you can’t predict upfront who’s going to react in what way, so that can be a complication.” Professional-15, genetic clinician
Unintended consequences following RGS results	
Expectations are high and the diagnosis maybe devastating	Q5: “Even if they do get a diagnosis, that diagnosis might not change anything but actually it might be a little bit of a death sentence and that is horrendous, like, for parents to have such hope and optimism and for that to be turned into something so negative, like, saying ‘oh we actually do have a diagnosis and I’m sorry it’s not good news’” Professional-7, medical doctor trainee, consenter for RaPS
Devastating diagnosis delivered before a chance to bond with the child	Q6: “Getting a devastating diagnosis before you’ve had the chance to bond with the child for instance, you can imagine maybe very difficult for a family to contend with. Whereas receiving a devastating diagnosis after a child has had a period of showing no progress or deteriorating and it being a much more slower realisation that actually things are not right, it maybe that a diagnosis at that point is something that’s easier for their family to contend with, rather than it being a real shock in the face of a child that’s unwell but hasn’t had a period of time, you know, to get to that point.” Professional-18, genetic clinician
Findings may bring additional problems to consider and concerns about the future	Q7: “They’ve got a young baby and actually maybe the outcome is going to be terrible whatever, maybe they don’t need their life sort of invaded by another set of things to think about, when actually the baby’s heart’s not working and in fact that’s the most important thing that day, and in fact maybe sadly that will be the thing that means the child doesn’t survive. Then to have us crowding in and coming in with another set of problems, which might for example then bring news which is oh by the way, the risk in another baby is high, that wasn’t something they were having to deal with at that point” Professional-15, genetic clinician
Partial, uncertain or no findings may add to anxiety and worry	Q8: “I haven’t got a reason why this happened, I’m always gonna like—want to get to the bottom of it.” Parent-10, child not diagnosed, VUS reported Q9: “Still a bit of anxiety because [Name] has so many other things. It would have just been nice that there was something that unified all of his issues” Parent-8, child partially diagnosed with STING-associated vasculopathy with onset in infancy
Disappointment and frustration when there is no diagnosis and no other tests to try	Q10: “They wanted to know and they were disappointed that we didn’t know…And it is tough because the next question is obviously what do we do next and, at the moment, the answer is ‘well nothing because I don’t have any wider testing to offer, we are just going to have to wait and see how this baby does and what becomes available in future’.” Professional-1, genetic clinician

#### Unintended consequences following RGS results

For parents who had a diagnosis identified for their child through RGS, professionals raised concerns about the impact on parents of a devastating diagnosis that would “shatter” their hope (Table 3: Q5) and how such a diagnosis for a newborn could negatively impact on bonding between parent and child (Table 3: Q6). Professionals also discussed the possibility of RGS results bringing additional problems to consider, including concerns about future children at a time, when parents are focused on the child’s current illness (Table 3: Q7).

For parents who received uncertain, partial or no findings for their child, participants described ongoing worry about the possible cause of the child’s illness (Table 3: Q8 and Q9) and some professionals noted that uncertain or no findings could “heighten the anxiety” for parents. There may also be disappointment and frustration for parents when there is no diagnosis and no other tests to try (Table 3: Q10).

### Counselling and decision-making in critical care settings

#### Challenges for informed decision making

Parents spoke of being incredibly stressed and tired at the time of being offered RGS (Table 4: Q1). Multiple teams are frequently involved in the child’s care and parents are asked to make many different complex decisions (Table 4: Q2). In addition, some parents found it difficult to accept that their child was unwell and were primarily focused on “medicines and surgery” (Table 4: Q3). Several professionals commented that the setting itself made conversations difficult and a quiet room is often not an option in ICUs. Overall participants felt that the situation made it very difficult for parents to give “due attention” to the details of RGS (Table 4: Q4). Several professionals commented that parents have been through so much they just want an answer, and parents all said that they would have done anything to help their child (Table 4: Q5). Accordingly, parents acknowledged making rapid decisions to have RGS (Table 4: Q6) and made it clear that they had not been concerned about issues such as data access and insurance (Table 4: Q7). One genetic professional observed that parents were less likely to refuse genetic testing in critical care settings as concerns about data security, are overridden by the child being so ill.

**Table 4:** Counselling and decision making in acute settings.

Topic	Illustrative quotes
Challenges for informed decision making	
Parents are made vulnerable by stress and lack of sleep	Q1: “That just him being ill there, took up all of our energy, yeah. It was confused, the life—it sucked everything out of us.” Parent-6, child diagnosed with Sotos syndrome
So much is going on when your child is in critically ill	Q2: “So it has happened that I’m doing the consenting [for RaPS] and there are like two teams waiting for me to finish to speak to these parents. Because they are going for, to the theatre and at the same time someone is to consent them for something else, and at the same time they need to go for an urgent MRI” Professional-4, genetic clinician
Immediate focus is surgery and medicine	Q3: “It’s a difficult thing to accept that your child is unwell or has a condition, so your immediate focus is on I suppose surgery and medicine and it was so difficult at the time perhaps to accept the testing side of it. But it’s got to happen to, it’s got to go hand in hand doesn’t it?” Parent-2, child partially diagnosed with Blepharophimosis, ptosis, and epicanthus inversus syndrome
Difficult to give full attention to testing decisions	Q4: “Normally the people that you’re approaching are in a terrible situation, intensive care, so I don’t think they’ve got the mind space for it.” Professional-2, non-genetic clinician
Parents would do anything to help their child	Q5: “I think we would have done anything to find out what was going on.” Parent-3, child diagnosed with Joubert syndrome
Parents make quick decisions to have RGS	Q6: “I said yes straightaway! I’ll say yes to anything, anything that might help” Parent-4, child not diagnosed
Parents put concerns such as data access and insurance to one side	Q7: “And yeah, we spoke about like you know data confidentiality, protection, all that stuff, ‘cause I was concerned about that with all the stuff about Google and 23andMe and everything, so that was a whole—and again, even if we had concerns, we weren’t going to do anything about it because we wanted to know what was going on with our daughter.” Parent-3, child diagnosed with Joubert syndrome
Decision making about secondary findings	
Parents want information about actionable secondary findings	Q8: “I could do a test and realise that something that’s going on with my future and I could start treating now—why not? I was really happy to do this test.” Parent-7, child diagnosed with Holt–Oram syndrome
Secondary findings present a difficult decision for some parents	Q9: “There was part of you that oh I just don’t want to know anything, if there’s nothing wrong with me. And there was another part of you that thought well, maybe I need to know, because this could be something that could affect me or the children in the future. And it’s probably the toughest part of that actually, in all honesty, but I think yeah, it was right down to the wire, and then we both said no, let’s just do it and we’ll go the whole hog. And if they find something at least we know.” Parent-11, child not diagnosed
Secondary findings not foremost in parents mind at this time	Q10: “I mean we mention it but I suppose if you’re thinking about some of the kids that we’re talking about you know, they’ve just come off a prolonged three weeks on intensive care. They’ve been told their child’s going to die. The secondary findings are not really foremost in their mind at the moment.” Professional-5, non-genetic clinician Q11: “You agreed to [secondary findings], but did you have any like concerns afterwards, thinking, oh do I really want to know about this? No, because at the time, we were living day to day—we were living in a day to day world. We didn’t know whether he was going to live or die.” Parent-6, child diagnosed with Sotos syndrome
Offering secondary findings in critical care settings requires additional considerations to safeguard families	Q12: “[Offering secondary findings] needs to be handled carefully particularly in that context where they might make a different decision in the acute setting compared to the sort of less stressed clinic setting. So I think we just need to be careful that we’re safeguarding families appropriately…I just think maybe—this is only my own theory—but potentially they’d be less bothered about that or give less thought to it [because the focus is] on the child.” Professional-8, non-genetic clinician Q13: “I do wonder whether a lot of people just tick that box without really thinking.” Parent-1, child diagnosed with Joubert syndrome
Parent reflections on pre-test counselling	
Information needs met and enough time to decide	Q14: “So it just seemed like a—yeah no brainer to go down this route. So yeah, but it was a lot to take in at the time. But like I said, it was all explained so many times, that we fully understood it, yeah…I think we were given enough time to take it on board. It wasn’t a case of just thrust in our face and making a decision, we need to know now. We had a good week to kind of go through everything and talk about it.” Parent-11, child not diagnosed
Written information is important	Q15: “If it’s with me, I will just think that I want genetic testing or not. If yes, then I would think OK, I want this genetic testing, that’s fine. So all the details, I can read it later on. But I’m not very ready to listen all the details right now, but that’s fine.” Parent-9, child diagnosed with multiple congenital anomalies-hypotonia-seizures syndrome
Expectations are high—care needed to convey limitations and possibility of uncertain or no results	Q16: “To be honest [a negative result] wasn’t something that I had thought about, thinking about it now. Yes, it, yeah, it wasn’t something that really crossed my mind ‘cause I think this was kind of our last hope to figuring out what this was, and/or getting the confirmation of what the doctors were thinking. So I hadn’t thought of the implications of had it come back negative.” Parent-3, child diagnosed with Joubert syndrome Q17: “They are hopeful and they’re saying OK someone will give me an answer, when everyone comes and looks at the baby and says I don’t know what this is—so they just put all their hopes on us, and then I guess we need to kind of stress the fact, highlight they may not get anything.” Professional-4, genetic clinician

#### Decision making about secondary findings

Parents were generally positive about being offered actionable secondary findings and were grateful for the opportunity to gain this information (Table 4: Q8). One non-genetic professional commented that “the feeling I get from the interactions we have with the families is that most people still want to know actually.” Most parents described readily agreeing to secondary findings, however, one parent said deciding about secondary findings as the most difficult part of the decision around RGS (Table 4: Q9). Participants noted that secondary findings would not be foremost in parents minds when consenting as their focus is obtaining a diagnosis for the child’s illness (Table 4: Q10 and Q11) and offering secondary findings in ICUs requires additional considerations to safeguard families (Table 4: Q12 and Q13). A parent with a secondary finding of a *BRCA* variant commented that she consented to secondary findings “not really thinking through” the implications, such as the possibility of sharing genetic information that maybe relevant to other family members; “I took that decision without really realising the impact it might have on other people.”

#### Parent reflections on pre-test counselling

Parents described the time of making a decision about RGS as a “whirlwind” or “blurred”, however, most were positive about pre-test counselling, saying they had enough information and did not feel rushed to make a decision (Table 4: Q14). When asked to recall the discussion, ten parents (of 11 interviewed) could describe some key aspects, such as looking at parent/child DNA; turn-around time; secondary findings maybe revealed; may not receive a diagnosis from the test. When asked what additional information would be helpful, parents highlighted the need for simple information in multiple languages with clarity on “what exactly they’re testing for and what they’re not testing for”. Written information was particularly important so that parents could return to it at a time when they could be more focused on the details of the test (Table 4: Q15). Some parents commented that they had not considered the possibility of a negative result (Table 4: Q16) and professionals noted that expectations for finding a diagnosis were high and care should be taken not to raise hopes (Table 4: Q17). Whilst being seen by multiple teams and being told of many possible potential diagnoses caused confusion and frustration for parents, benefits of multiple care teams, such as being given information from two different perspectives were also highlighted.

### Challenges for service delivery as RGS for critically ill children moves into routine clinical care

Professionals were asked about the key challenges and educational needs for delivering a clinical service. See Supplementary Materials for illustrative quotes.

#### Key factors for effective service delivery

Delivering RGS in ICUs was described as being complex, as “it requires a lot of organisation, a lot of people to be involved to ensure that the process is smooth from beginning to end”, and labour intensive with involvement required from many staff groups to decide eligibility for testing, undertake counselling and consent, perform laboratory and bioinformatics analyses, and interpret results. Clear care pathways were viewed as important to ensure rapid turn-around with standardised requirements for phenotyping to aid interpretation of sequencing results. Collaborative working between clinical and laboratory genetics and the child’s specialty teams was highlighted as essential by many professionals, with good communication between these teams and regular MDT meetings highly valued. The RaPS study had promoted effective collaborative relationships between clinical and laboratory teams, and there was some concern that if testing was performed remotely those beneficial interactions would be lost.

#### Who should be offered RGS?

The strict eligibility criteria used in the RaPS study were supported, with professionals suggesting that there were more children that may benefit from the test, but accepting that costs, capacity and resources will limit numbers. It was noted that there is no good data on which groups of patients will benefit most. Some professionals highlighted the value of offering RGS “as early as possible just to avoid testing and retesting”. They acknowledged that achieving a balance between ruling out obvious conditions and doing many unnecessary tests before offering RGS was a key challenge for clinical implementation. The value of offering microarray prior to RGS was raised as a way to quickly check for a range of conditions and the wait for these results could be useful; “just that pause, have we got all the information about the baby, how urgent is this situation”. In addition, some professionals noted that it was becoming less acceptable for children to not have a genetic diagnosis and there would be pressure to test children who would have previously been managed based on a clinical diagnosis.

#### Who should conduct pre- and post-test counselling?

In the RaPS study, the genetics team led the delivery of RGS, but professionals felt that, as testing became part of mainstream care, non-genetics clinicians could offer these tests if they have had appropriate training. However, although some clinicians felt comfortable requesting genetic tests for known conditions, many felt that the genetics team should initially lead a RGS service, particularly with respect to deciding eligibility, interpreting variants and counselling for complex or uncertain findings. Several, genetics professionals highlighted the importance of understanding the limitations of RGS, particularly noting concerns about not understanding that a negative result did not rule out the possibility of a genetic condition being present and “that you don’t assume that a variant of unknown significance is causative until you have the evidence to show that it is”. Conveying the value of comprehensive phenotyping for guiding sequencing interpretation was also noted. In addition to training in genetics and genomics, it was felt that non-genetics clinicians will need to build their confidence and skills in pre- and post-test counselling.

## Discussion

As RGS is increasingly used clinically, consideration of parent and professional perspectives around its usefulness, the potential for unintended harms and challenges is key for delivering an efficient and appropriate clinical service. This is particularly timely as RGS for critically ill children was introduced in October 2019 within the UK Genomic Medicine Service. An important strength of our study is the integration of the views and experiences of parents and health professionals who notably held largely consistent views. Benefits of RGS described in published studies of clinical utility [4–13], including guiding treatments and transplant decisions, preventing unnecessary interventions and initiation of palliative care were valued by professionals and parents in our study. However, our qualitative research has allowed us to gain a more nuanced understanding of these clinical benefits and appreciate the psychosocial benefits for parents which included relief, reassurance and the opportunity for greater certainty.

Similar to studies conducted with parents of children having RGS in the NICU [15], parents articulated concerns that the offer of RGS was confronting as being offered the best testing available reinforces the seriousness of the child’s illness. In addition, we found that concerns that RGS could reveal a devastating diagnosis and that the possibility of multiple different diagnoses was burdensome, with parents worrying about conditions that did not turn out to be the diagnosis. Professionals were concerned that RGS results could shatter parent’s hopes and negatively impact on bonding with neonates, an issue discussed in the ethics literature [24, 25]. Ongoing anxiety and frustration for parents when there are uncertain findings or no diagnosis were also highlighted. Many of these concerns would be relevant to any genetic tests offered in this setting, however, RGS differs in a number of ways, including the broad scope of the test, the rapid turn-around time and the potential for uncertain findings. When RGS is offered in critical care settings, parents may need additional support while waiting for the outcomes of testing and could benefit from established plans for clinical follow-up [12].

ICUs are unquestionably a difficult environment for parents who have increased stress levels, anxiety, anger and depression, compared with those with children on other wards [26, 27]. Supporting informed decision making has been highlighted previously as a key challenge for offering RGS in acute care settings [12, 24]. We found that the vulnerability of parents in this setting was clear and parents spoke about doing anything to help their child. They put aside concerns around data sharing and insurance, which are the commonly cited reasons for declining genomic sequencing in other settings [28]. When reflecting on being offered RGS parents felt the choice was straightforward, they had sufficient information and time to make a decision and none reported regretting their decision, which are all indicators of informed decision making. These findings reflect research on RGS in the NICU that concluded that parents were not naïve about risks of RGS and weighed up benefits and concerns when making decisions [15]. In addition, research on consent for general medical procedures in an ICU [29], found parents wanted to be informed and take an active role in decision making despite the stress of the environment.

Our findings suggest that to support informed decision making, pre-test counselling should provide parents with realistic expectations, including the impact of a diagnosis that maybe life-limiting, a rare diagnosis, the possibility of not receiving a diagnosis, or of uncertain findings. It also needs to be clear that diagnosis may not impact on care. The implications of secondary findings for the individual and other family members also needs discussion before parents decide. As observed previously, some parents preferred to defer decisions on secondary findings, while others opted in so they had the chance to address health concerns [30]. We also found that parents, particularly those without a diagnosis, may want more information on certain topics, such as which genes had been tested and whether reanalysis would be available to them in the future. This is in keeping with studies of RGS offered in less critical situations [31, 32] and reflects suggestions that patients want additional information and communication throughout the process of GS [33] and that there maybe a need to revisit consent conversations over time [34]. Access to written information that would allow parents to return to the information when they felt able was also considered important.

Highlighted challenges for service delivery centred on issues arising from RGS being complex and labour intensive, with clinical implementation requiring sufficient appropriately trained staff to facilitate a rapid turn around. Comparable findings from other studies highlight the potential for additional pressures on staff and burdens for laboratories [10, 20]. These issues need consideration as RGS shifts from small scale research to wider clinical implementation. All professional groups valued cross-discipline collaborative working when delivering RGS, highlighting the benefits of multidisciplinary team meetings to triage patients and interpret results. As with other studies in acute [18] and more routine settings [20, 35, 36], we noted that as GS moves to mainstream clinical practice [37], a growing role for non-genetic professionals was recognised. Additional training is needed alongside support from genetics colleagues for decisions on eligibility, interpretation of results and counselling. A potential role for genetic counsellors in delivering RGS in ICUs has been suggested, as they are well placed to support the information and emotional needs of parents [10, 12, 30], and this should be explored as we deliver RGS in the UKs Genomic Medicine Service.

### Limitations

A key limitation to the generalisability of this study is that participants were a small and self-selected group of parents and professionals and responder bias maybe an issue. Parents of critically ill children have been in a stressful and emotional situation because of their child’s illness and it maybe difficult to untangle the experience and psychosocial impact of being offered RGS from the impact of the illness or the diagnosis that was made. The age of the child and the type of condition diagnosed or a lack of diagnosis will also be factors. Also the retrospective nature of data collection and the time that had passed since being offered RGS (1–4 years) may have created a recall bias for parents. In addition, the parents we interviewed all chose to have RGS and the positive views about the test may reflect “choice-supportive bias”, whereby a person recalls their choice more positively simply because it was the choice they had already made [38]. Combining the findings of the parent and professional interviews, as we have done in this study, could decrease the impact of the individual cognitive biases of the parents we interviewed as the professionals have provided a wider perspective on how parents view RGS. However, further research is required with a prospective design that can capture parent’s thoughts on their experiences and decision making close to the time of being offered RGS.

It should also be noted that the study was conducted at a single centre with expertise in rare diseases and an onsite genomic laboratory. As a result, the professionals we interviewed may have different views to professionals in other settings. Another consideration for generalisability is that although the parents we spoke to had a range of outcomes from RGS, we did not interview any parents whose child died, any parents that declined RGS or any that had older children who were in intensive care because of ongoing chronic symptoms and these groups may have distinct views.

Another potential limitation of the study was that in all but one case parents opted for a telephone interview, generally for practical reasons such as constraints in their availability due to work or child care. Although preliminary interactions and non-verbal communication which can help to build rapport are precluded with a telephone interview, direct comparison of telephone and face-to-face interviews suggest that the quality and richness of the data collected is equivalent [39]. In addition, participants value the practical benefits of being able to participate in an interview by telephone and may feel more relaxed and comfortable discussing sensitive subjects [40, 41].

## Conclusion

Healthcare professionals and parents with direct experience of RGS for critically ill children have largely positive views of RGS in this setting. Attentive pre-test counselling is needed to support informed parental decision making. Many parents need continued support while waiting for, and after receiving test results. As this test moves to clinical practice nationally in the UK, further empirical research evidence exploring the experiences of parents and professionals is needed to inform policy and practice. As RGS enters clinical practice it will be important to explore how robust informed consent processes can be supported outside of a research context, with a particular focus on secondary findings. In addition, an in-depth exploration of parent’s ongoing communication and support needs is warranted with research needed into the development and evaluation of decision aids that can support decision making specifically in critical care settings. It will also be important to examine the effectiveness of training in building both knowledge and confidence in offering RGS for non-genetic clinicians.

## Supplementary information

Supplemental Tables
