# Supplementary material for: Delivering genome sequencing for rapid genetic diagnosis in critically ill children: parent and professional views, experiences and challenges
Source: Eur J Hum Genet. 2020 Jun 19;28(11):1529–40. doi: 10.1038/s41431-020-0667-z (PMC7575551; doi:10.1038/s41431-020-0667-z)
Supplement: Supplementary file 1 — Supplemental Tables [file 41431_2020_667_MOESM1_ESM.docx]

**Supplementary materials**

Health professional views on the key challenges for delivering a rapid GS for critically ill children as a clinical service

**Table 1:** Key factors for effective service delivery

| **Topic** | **Illustrative quotes** |
| --- | --- |
| Rapid GS is labour intensive | “You have to take a person almost dedicated to doing it. At the moment we’re only running one single trio a week, so if you wanted to do more we could property do two trios a week fairly comfortably, it would take a bit of work and may take a bit more hands on by someone else helping in terms of analysis. It just takes a lot of time.” Professional-9, scientist |
| Staff numbers and availability needs consideration | “I still feel that definitely you need a larger infrastructure that will include more bodies in the service, definitely more geneticists, more clinical scientists… the way I have seen it is that you need more people there that can do these tests.” Professional-12, non-genetic clinician |
| Clear care pathways and a consistent approach | “I think it makes sense to have a very clear pathway to consent because on the back of the consent, the other things that need to be done is things like there needs to be information filled in and completed about appropriate phenotypic terms and panel design and all of those sorts of things... if you’re offering this across the board to clinicians there needs to be some consistency of approach and understanding of that process which not everybody is comfortable or familiar with.” Professional-18, genetic clinician |
| Good communication between teams is needed | “You need to have regular meetings. You need to have good communication between teams and ideally teams getting involved right from the beginning” Professional-18, genetic clinician |
| MDT’s and collaborative working are important | “And then you probably need good MDTs that will involve clinicians, not only clinical geneticists… [but also] the lead specialty for each child.” Professional-12, non-genetic clinician |
| In-house testing facilitates strong relationships with clinicians and laboratory teams – may change with a national service | “we have a very good service, very good relationship with our lab team here and there’s always a concern that you're going to lose some of that expertise if it’s been shunted outside… And there’s obviously a big change in terms of where the analysis is being done on the bioinformatics and who’s going to be analysing what. And actually the human interaction with those people who are doing that analysis is really helpful and really important, which I would worry slightly if things are done remotely because you don't have quite the same sitting round the table interaction that sometimes is really helpful.” Professional-8, non-genetic clinician |

**Table 2:** Who should be offered rapid GS and when?

| **Topic** | **Illustrative quotes** |
| --- | --- |
| Support for strict eligibility criteria | “I think you need somebody strictly looking at it as a resource… I think it’s really important that there’s tight criteria… Are you really going to do something differently here? Why are you not waiting eight weeks for this result because a routine lab can send this through in eight weeks? I would say it has to be very, very clear cut what you’re doing and the original remit was life threatening disease, trying to make decisions that influence, have big influences on life decisions. I think that’s what it should be. I mean withdrawing care on PICU is a good example, if that makes things easier for families around that, having a diagnosis, of course it’s going to. I think going for a bone marrow transplant is a similar life changing thing.” Professional-5, non-genetic clinician |
| Number of tests is dependent on lab capacity and costs | “Because we had limited resources, it cost about £7,000 to do a whole genome sequencing and so we really wanted to make this available to whomever could benefit from it the most, but if it is to be offered as a service, I think everyone should have an equal opportunity but depends on what we can afford, so it’s really down to money, yeah.” Professional-14, scientist |
| More children could benefit from rapis GS, but research is needed to show which children will benefit from rapid ES / GS | “So there are criteria, but it’s really difficult because the availability of testing in RaPS has been very small in numbers. So the number, at *[Hospital 1]*, the number of patients who are on the ward at any given time who could potentially benefit from it is very much larger than the children who you can actually sequence. So it’s really difficult and there’s not perfect data, there’s not good data at the moment on what proportion children on intensive care say would benefit from an approach like RaPS.” Professional-15, genetic clinician |
| Avoid testing and re-testing | “my personal opinion would be particularly for this cohort of patients where they are quickly deteriorating, they have a very broad genetic differential diagnosis, so I think the clinical team discusses them very quickly because they need intervention quickly. And so if they know that the testing will take longer, I think it’s best to adopt it as early as possible just to avoid testing and retesting.” Professional-14, scientist |
| Targeted test or ES / GS | “But broadly I think, in the right child, it can be the right first line test, but you, one of the challenges of rolling out[rapid GS] will be at the beginning it’s you know, it still needs expertise in picking the right patient and making sure there’s not a better test.” Professional-15, genetic clinician |
| Microarray before ES / GS | “I think most patients would probably have a microarray around referral time. Just because it’s sort of a quick and easy, you know it’s essentially an alternative whole genome test really isn’t it?… But otherwise I feel like it should be the first port of call because you’re just testing as much as you can initially, yeah.” Professional-10, scientist |
| Increasing pressure to have a genetic diagnosis | “so some clinicians have for many years discussed serious conditions based on the clinical features and they're… content to say, to manage a child based on the clinical diagnosis and I guess that’s less – there are fewer people who are going to do that because they don't want to be caught out, whether medical legally, whether anything, so there will be more pressure to have done all we can to confirm diagnosis in children who have got a collection of abnormalities or malformations or presentations.” Professional-17, genetic clinician |

**Table 3:** Supporting the growing role of non-genetics clinicians in offering rapid GS

| **Topic** | **Illustrative quote** |
| --- | --- |
| Genetics team currently lead on counselling and consent | “I’ve – in the room, and mentioned a genetic test we can send off, but it’s normally the geneticist, I say then “The genetics team will come and explain more about this to you”. I’d be surprised if many of my colleagues don’t do that.” Professional-13, non-genetic clinician |
| Non-genetics professionals have a growing role in offering ES / GS | “I think there is a certain fear of the unknown. [genetics is] revolutionising whole areas of medicine, it is something that we’re going to have to be aware of and find a place for, and not have to rely on clinical genetics for, you know, it should become more and more part of, you know, embedded in our practice… It’s difficult. But I think yeah, it will be an increasing part of our roles.” Professional-19, non-genetic clinician |
| Non-genetics should offer ES / GS with training and support | “I think if people have the clinical acumen and understanding of what it the disease is, and they know which test to choose, then I see no reason why they should not be able to request for that.” Professional-11, non-genetic clinician  “we’re pretty much all of us are sending off gene tests now, from virtually any aspect of certainly paediatrics, any discipline. So I guess you just, people just need to make sure they're trained and have been to the appropriate level training” Professional-3, non-genetic clinician |
| Many non-genetics professionals are not yet comfortable to offer ES or GS | “I think there are probably a lot of paediatricians who aren't really sure and would feel uncomfortable having to do it on their own, so I think there probably needs to be more geneticist or there needs to be more training around the physicians, paediatricians who are going to do it.” Professional-2, non-genetic clinician |
| Awareness of when to seek help | “I guess if people don't feel comfortable that they shouldn't be doing it, that they should just say that it needs to go back to genetics, if paediatricians, the physician who first gets the result isn't comfortable relaying it then they probably need to be aware of that and step out.” Professional-3, non-genetic clinician |
| Geneticists will lead service delivery for now | “so I think there is going to be this transition phase where people start to understand the power of whole genome sequencing and understand that it can be useful and then they’ll start to understand that they need to understand about genetics. And, in the meantime, I guess the geneticist will stand in the gap.” Professional-1, genetic clinician |
| Genetics support needed for deciding eligibility of cases | “Doesn’t necessarily have to be genetics consenting and things but needs to the involvement from the, even if it’s in the discussion level and making sure that all the right documentation is there, and there needs to be some involvement and awareness of cases that have been there and the appropriateness of accepting cases. And so don’t necessarily need to be the ones consenting but they do need to be part of that process.” Professional-18, genetic clinician |
| Genetics involvement important for interpreting and discussing unusual and uncertain findings | “I think it depends on how each healthcare professional feels, you know, how they feel about it and whether they feel comfortable in discussing, you know, especially if you’re dealing with some kind of rare condition then I think it’s best to come from a geneticist. However, if you are talking about testing for the known genes for example in the future, that you know what’s the likely outcome going to be, then the clinician may be very happy because we’ll be dealing with these known genes anyway. But I think currently with the current situation I would probably think that it’s 50/50, but I would more say more of a genetic involvement at this point in time knowing that you might be dealing with some really unusual findings.” Professional-16, non-genetic clinician  “I think it’s really important to have, you know when you’re actually kind of delving down into the interpretation of variants and the dissemination of those results and talking about the likelihood of diagnoses being made and how equivocal those can be. I think it’s really useful to have clinical genetics expertise to kind of convey that uncertainty.” Professional-10, scientist |

**Table 4:** Professional’s suggestions for training content to upskill non-genetics clinicians

| **Topic** | **Illustrative quotes** |
| --- | --- |
| Basic principles of the technology and the limitations | “for any test, you know, what is it able to do, what’s it not able to do and what else might it throw up, as basic ideas... I would need to understand the technology and the technical limitations as well.” Professional-8, non-genetic clinician |
| Differences between available tests | “definitely need to understand the different between array, targeted molecular testing for a particular gene and whole exome and whole genome.” Professional-4, genetic clinician |
| Interpretation of results | “this is what we’ll feedback to you and this is how you look at interpretation and this is who you go to if you're not clear about interpretation.” Professional-1, genetic clinician |
| Variants of unknown significance | “how important it is that you don't assume that at a variant of unknown significance is causative until you have the evidence to show that it is.” Professional-1, genetic clinician |
| A negative result does not rule out a genetic condition | “Yes, so that would be the main problem, if we don’t find anything, and if people after not think that this patient has had whole genome sequencing and we’re not offering anything else, we’ve finished the genetics.” Professional-4, genetic clinician |
| Importance of phenotyping for interpretation | “the importance of the phenotyping informing the analysis that’s done” Professional-18, genetic clinician |
| Skills and information for counselling and consent | “if I wanted to be trained to be able to take consent formally, it would be knowing what process families would go through, how information’s going to be fed back, yeah, all those things.” Professional-8, non-genetic clinician  “I do think people need to know how to consent properly and need to discuss things like know how to discuss things like secondary findings” Professional-18, genetic clinician |

**Table 5:** Professional’s suggestions for training delivery to upskill non-genetics clinicians

| **Topic** | **Illustrative quotes** |
| --- | --- |
| Embed genetics in medical education | “genetics education needs to be ingrained in medical education increasingly going forward” Professional-18, genetic clinician |
| Competency | “[there are] all sorts of things that you need to kind of keep up a basic level of competence, so perhaps there should be a competency for anybody that might be giving genetic results” Professional-3, non-genetic clinician |
| Mandatory training | “individuals in a hospital like GOSH where the patient population is so complex and there’s a huge amount of rare disease here, that needs to be, my feeling is it needs to be part of mandatory training” Professional-18, genetic clinician |
| Education from the genetics team | “a big role of the Genomic Medicine Centres, and of the genetic counsellors and doctors, is that education role, so that it is something that can be safely and well delivered outside just those specialists.” Professional-15, genetic clinician |
| Teaching through practice – genomics champions | “people teaching through practice and you know because, these concepts of genomic champions in different specialties being the sort of, the standard bearers, and then people learning by osmosis.” Professional-15, genetic clinician |
| Ongoing training | “Health Education England have set up all of these MScs. which are open to all sorts of different people and that’s a way of doing it, but it needs people to be interested enough to sign up for them” Professional-15, genetic clinician  “I think probably is an ongoing process to make sure you keep informed” Professional-13, non-genetic clinician |
| Study days and workshops | “I think having study days would be helpful.” Professional-6, non-genetic clinician  “So for somebody like my generation, I think some of those workshops and things, just getting up to speed in the language would be very helpful” Professional-2, non-genetic clinician |
